# Modulation of Aneuploidy in *Leishmania donovani* during Adaptation to Different *In Vitro* and *In Vivo* Environments and Its Impact on Gene Expression

**DOI:** 10.1128/mBio.00599-17

**Published:** 2017-05-23

**Authors:** F. Dumetz, H. Imamura, M. Sanders, V. Seblova, J. Myskova, P. Pescher, M. Vanaerschot, C. J. Meehan, B. Cuypers, G. De Muylder, G. F. Späth, G. Bussotti, J. R. Vermeesch, M. Berriman, J. A. Cotton, P. Volf, J. C. Dujardin, M. A. Domagalska

**Affiliations:** a Molecular Parasitology, Institute of Tropical Medicine, Antwerp, Belgium; b Wellcome Trust Sanger Institute, Hinxton, Cambridge, United Kingdom; c Charles University, Prague, Czech Republic; d Unité de Parasitologie Moléculaire et Signalisation, INSERM U1201, Institut Pasteur, Paris, France; e Mycobacteriology Unit, Institute of Tropical Medicine, Antwerp, Belgium; f Advanced Database Research and Modelling (ADReM), Department of Mathematics and Computer Science, University of Antwerp, Antwerp, Belgium; g Molecular Cytogenetics and Genome Research, Department of Human Genetics, KU Leuven, Leuven, Belgium; h Department of Biomedical Sciences, University of Antwerp, Antwerp, Belgium; University of Oxford

**Keywords:** *Leishmania*, aneuploidy, gene dosage, genomics, life cycle

## Abstract

Aneuploidy is usually deleterious in multicellular organisms but appears to be tolerated and potentially beneficial in unicellular organisms, including pathogens. *Leishmania*, a major protozoan parasite, is emerging as a new model for aneuploidy, since *in vitro*-cultivated strains are highly aneuploid, with interstrain diversity and intrastrain mosaicism. The alternation of two life stages in different environments (extracellular promastigotes and intracellular amastigotes) offers a unique opportunity to study the impact of environment on aneuploidy and gene expression. We sequenced the whole genomes and transcriptomes of *Leishmania donovani* strains throughout their adaptation to *in vivo* conditions mimicking natural vertebrate and invertebrate host environments. The nucleotide sequences were almost unchanged within a strain, in contrast to highly variable aneuploidy. Although high in promastigotes *in vitro*, aneuploidy dropped significantly in hamster amastigotes, in a progressive and strain-specific manner, accompanied by the emergence of new polysomies. After a passage through a sand fly, smaller yet consistent karyotype changes were detected. Changes in chromosome copy numbers were correlated with the corresponding transcript levels, but additional aneuploidy-independent regulation of gene expression was observed. This affected stage-specific gene expression, downregulation of the entire chromosome 31, and upregulation of gene arrays on chromosomes 5 and 8. Aneuploidy changes in *Leishmania* are probably adaptive and exploited to modulate the dosage and expression of specific genes; they are well tolerated, but additional mechanisms may exist to regulate the transcript levels of other genes located on aneuploid chromosomes. Our model should allow studies of the impact of aneuploidy on molecular adaptations and cellular fitness.

## IMPORTANCE

Aneuploidy is usually detrimental in multicellular organisms, but in several microorganisms, it can be tolerated and even beneficial. *Leishmania*—a protozoan parasite that kills more than 30,000 people each year—is emerging as a new model for aneuploidy studies, as unexpectedly high levels of aneuploidy are found in clinical isolates. *Leishmania* lacks classical regulation of transcription at initiation through promoters, so aneuploidy could represent a major adaptive strategy of this parasite to modulate gene dosage in response to stressful environments. For the first time, we document the dynamics of aneuploidy throughout the life cycle of the parasite, *in vitro* and *in vivo*. We show its adaptive impact on transcription and its interaction with regulation. Besides offering a new model for aneuploidy studies, we show that further genomic studies should be done directly in clinical samples without parasite isolation and that adequate methods should be developed for this.

## INTRODUCTION

Aneuploidy is usually deleterious in multicellular organisms (e.g., trisomy 21 or cancers in humans) but is tolerated and potentially beneficial in some unicellular organisms (1, 2). The best-studied models are fungi, especially *Saccharomyces* and *Candida*, in which aneuploidy can be encountered in natural isolates and in laboratory strains (1, 3–5). The phenomenon may concern more than one chromosome (6), and it was reported to promote adaptation to stress (6, 7). More specifically, in pathogenic fungi, it seems to contribute to virulence (8) and appears as an intermediate step during the development of drug resistance (9).

*Leishmania* (Protozoa, Kinetoplastida, Trypanosomatidae) species are parasites of high medical and veterinary importance and are emerging as a new model for aneuploidy. Several reports describe aneuploidy in *Leishmania*, in contrast to other members of the family Trypanosomatidae, like *Trypanosoma brucei* or *Trypanosoma congolense* (10, 11). Although the baseline somy is 2N, high levels of aneuploidy are found in all *Leishmania* species studied to date. Whole-genome sequencing (WGS) of 204 *Leishmania donovani* strains from the Indian subcontinent recently revealed tremendous interstrain diversity of karyotypes, with up to 22 polysomic (somy greater than 2) chromosomes out of 36 in single strains (12). Only chromosome 31 was tetrasomic in all 204 strains, as well as in other species, while only chromosome 25 remained disomic in all strains (11, 12). Furthermore, this variation also exists as mosaicism within strains: in the population of cells constituting a strain, fluorescence in situ hybridization (FISH) shows that individual cells have different somies for a given chromosome (13). Lastly, aneuploidy has been shown to play a role in the development of drug resistance, as in pathogenic fungi (14, 15).

The high interstrain and intrastrain diversity of karyotype in *Leishmania* organisms may play a major role in the biology and evolution of the parasite. On one hand, the fluctuations in somy allow the parasites to control heterozygosity and, as such, aneuploidy could participate in a novel parasexual model of reproduction (16). On the other hand, aneuploidy has been proposed to be one of the strategies used by *Leishmania* for modulating gene expression (14, 17). In the absence of transcription regulation at initiation through promoters (18), increased chromosome or gene copy number represents a solution for the parasite to increase transcript levels (14).

In the natural transmission of *Leishmania*, extracellular flagellated promastigotes proliferate in the midgut of sand flies, from where they are introduced into vertebrate hosts during the next blood meal. These promastigotes are taken up by phagocytic cells in the vertebrate, where they transform into amastigotes; these intracellular forms then transform back to promastigotes when taken up by a new sand fly. Considering the postulated major impact of aneuploidy on gene dosage and its potential functional implications, we investigate here (i) how *L. donovani* genome diversity and, in particular, aneuploidy evolves during the parasite’s life cycle and its adaptation to the different environments and (ii) what impacts the observed changes have on the transcriptome. We followed the whole-genome variation of different strains, using experimental infections of Syrian golden hamsters and sand flies to investigate *in vivo* amastigote and promastigote stages, respectively, and comparing them with *in vitro*-maintained parasites. We document an unexpected modulation of aneuploidy during the life cycle and dissect transcriptional impacts related to adaptation and regulation.

## RESULTS

### *In vitro* and *in vivo* dynamics of *L. donovani* genome.

**FIG 1:**
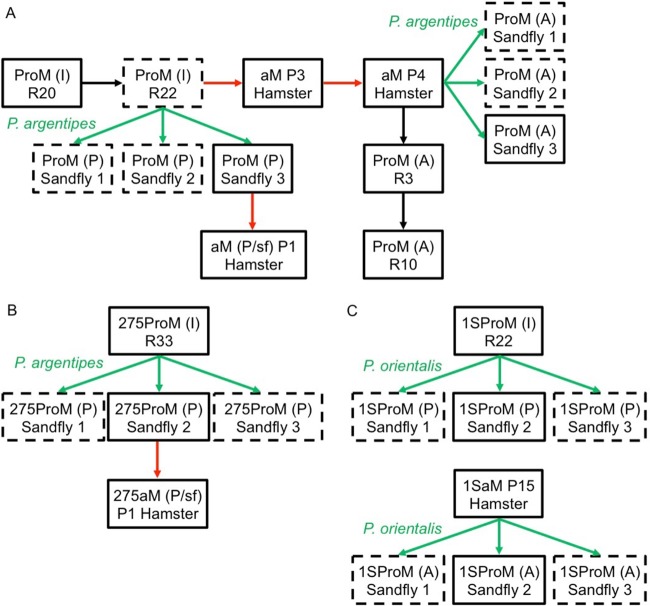
Flowchart showing the experimental histories of the 3 *L. donovani* strains, BPK282/0 (A), BPK275/0 (B), and Ld1S (C). Black arrows indicate *in vitro* passage (R, with the number of subinoculations since isolation from the patient or animal); red arrows indicate passage through hamster (P, with the number of passages in the animal); green arrows indicate a transmission through the natural vector named in green. Samples that were submitted to DNA and RNA sequencing are in solid-line boxes, while samples for which only DNA sequencing was performed are in dashed-line boxes. ProM, promastigotes; aM, amastigotes. Letters within brackets indicate the life stage and environmental conditions from which the parasites came, as follows: I, *in vitro* promastigotes; A, amastigotes from hamster; P, promastigotes from *in vitro* culture; P/sf, sand fly-originated promastigotes.

We tracked genome diversity by whole-genome sequencing (WGS) (sample details are in Table S1A in the supplemental material) at different levels (single-nucleotide polymorphisms [SNPs], indels, copy number variations [CNVs], and somy) during *in vitro* and *in vivo* maintenance of our reference BPK282/0 line. Briefly, the experiment started with *in vitro*-cultured promastigotes, and we compared (i) promastigotes reisolated from sand flies infected by cultured promastigotes, (ii) amastigotes during short-term and (iii) long-term adaptation to hamster, and (iv) promastigotes reisolated from sand flies infected by hamster-derived amastigotes and (v) during adaptation to *in vitro* culture of amastigote-derived promastigotes (Fig. 1A, see flowchart).

10.1128/mBio.00599-17.4 TABLE S1  Sequencing details from DNA-seq (A) and RNA-seq (B). (A) Displays sample name and sequence ID associated with the library preparation method and the use we made of each sample in the study. Depth details for each sample are also mentioned. (B) Details of each sample used for the transcriptomic analysis, including sample and sequence name and their respective mapping efficiency and sequencing depth statistics. Download TABLE S1, PDF file, 0.04 MB. Copyright © 2017 Dumetz et al. 2017 Dumetz et al. This content is distributed under the terms of the Creative Commons Attribution 4.0 International license.

When the nucleotide sequences of all samples were compared, we detected no local CNVs (Table S2A) or indels and only 5 heterozygous SNPs in the nuclear genome that changed in frequency during the experiment (Table S2B). Among these, 4 heterozygous SNPs on chromosomes 24, 32, and 25 were not present in cultured promastigotes but were detected only in the adapted amastigotes (aM P3 Hamster and aM P4 Hamster) (Fig. 1) and were maintained in the subsequent *in vitro*-grown and sand fly-derived promastigotes. We also observed the disappearance of a heterozygous SNP located in chromosome 35 in sand fly-derived promastigotes where promastigotes were used as the inoculum (Table S2B). No new SNPs were detected in the kinetoplast DNA (kDNA) maxicircle.

10.1128/mBio.00599-17.5 TABLE S2  (A) Local copy number variation of major amplicons during adaptation of BPK282/0 to different environments. Three loci (H locus, M locus, and chr6 “Yeti” amplicon) known to vary in copy number in natural populations (12) were analyzed. For each amplicon and the different genes present in the respective amplicons, we consider the average copy number per haploid genome. The chr6 “yeti” amplicon was observed so far only in ISC001 isolates of *L. donovani* (Highland parasites from Nepal, genetically very different from main population of the lowlands [12]) and never in the lowland parasites: in the current study, the locus remained clearly single copy in all life stages studied. The depth values are based on median haploid depths that are not affected by somy variations (see Text S1). In the table, the normalized standard deviations (Norm std.) and raw median depth (DEPTH) were given for assessing the quality and variability of depth of each sample. Library preparation N means Illumina Nextera, and this is shown because the depth variation tended to be different from those of other library preparations. (B) SNP frequency variation during adaptation of BPK282/0 to different environments. The table shows the number of reads for DNA-seq and RNA-seq. The ratio *x*/*y* means the reference base count *x* and the alternative allele count *y*. Only the SNPs varying in frequency over the course of the experiments, whose accuracy was also verified in Integrative Genomics Viewer (IGV), are shown. BPK282 is the reference strain from which we created the reference base count, and *Leishmania donovani* in the Indian subcontinent is known to have very few heterozygous SNPs (12). Download TABLE S2, PDF file, 0.1 MB. Copyright © 2017 Dumetz et al. 2017 Dumetz et al. This content is distributed under the terms of the Creative Commons Attribution 4.0 International license.

**FIG 2:**
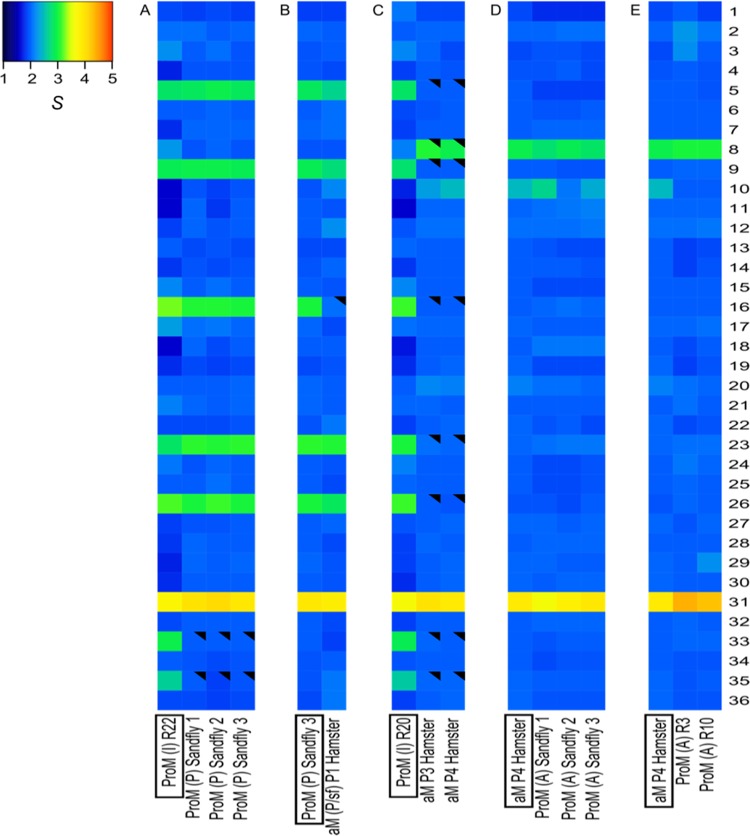
Dynamics of aneuploidy of *L. donovani* BPK282/0 during adaptation to different *in vitro* and *in vivo* environments. (A) Promastigotes reisolated from sand flies. (B) Amastigotes during short-term adaptation. (C) Amastigotes during long-term adaptation. (D and E) Promastigotes reisolated from sand flies infected by hamster-derived amastigotes (D) and during adaptation to *in vitro* culture of amastigote-derived promastigotes (E). Heat maps show median normalized read depths of chromosomes found within each cell population for each of the 36 chromosomes (*y* axis) and each sample (*x* axis). The color key shows the normalized chromosome read depth (*S*) and the distribution frequency. *S* ranges are as follows: monosomy, *S* < 1.5 (dark blue); disomy, 1.5 ≤ *S* < 2.5 (light blue); trisomy, 2.5 ≤ *S* < 3.5 (green); tetrasomy, 3.5 ≤ *S* < 4.5 (orange); pentasomy, 4.5 ≤ *S* < 5.5 (red). A filled black triangle in an upper right corner indicates a significant change of *S* (≥0.5, with a shift from one *S* range to another and a *P* value of ≤10^−5^) in comparison to the *S* value of the sample framed in a black box at the bottom.

In contrast with the overall high conservation of the nucleotide sequence of the parasite genome, we observed drastic changes in karyotype in the different life stages and/or environments. For each chromosome, the median somy value *S* within each sample, its corresponding median absolute deviation (MAD) across reads of that chromosome, and the statistical significance of differences in *S* values between samples were calculated as described in Materials and Methods (see Data Set S1A1 in the supplemental material); when referring to integer somy (di-, tri-, and tetrasomy), we followed the rules described previously (11, 12, 19). When we inoculated *in vitro*-adapted promastigotes (with aneuploidy affecting 8/36 chromosomes) into a sand fly and reisolated parasites at the late stages of infection (5 days in the fly, followed by 5 days of propagation in culture), we observed that the *S* values of chromosomes 33 and 35 decreased significantly, from 2.95 ± 0.62 to 2.00 ± 0.14 (average ± standard deviation of 3 sand fly samples) and from 2.64 ± 0.59 to 1.92 ± 0.11, respectively, while the rest of the karyotype remained unchanged (Fig. 2A; see also Table S3 and Data Set S1A1). Importantly, we observed identical karyotypes in promastigotes isolated from 3 independent flies, suggesting that these aneuploidy changes were not random (Data Set S1A1 and A2). Next, we inoculated the promastigotes isolated from one sand fly into hamsters, and after 2 months of infection, we analyzed the karyotype of the purified amastigotes [aM (P/sf) P1]. We found that the overall aneuploidy pattern of these amastigotes was similar to that of the inoculum, except for chromosome 16, for which *S* dropped from 3.03 ± 0.17 to 2.08, where the MAD could not be measured for aM (P/sf) due to insufficient depth of coverage. The aneuploidy changes were more pronounced between *in vitro*-adapted promastigotes and the derived amastigotes after 3 and 4 consecutive passages between hamsters, meaning, respectively, 12 and 19 months after the first inoculation of promastigotes. In promastigotes, the *S* values of 7 chromosomes (Table S3) ranged between 2.60 ± 0.45 and 3.16 ± 0.51, and they decreased significantly in amastigotes, ranging between 1.98 ± 0.07 and 2.06 ± 0.07. The *S* values of chromosome 31 remained in the range of tetrasomic chromosomes (3.5 ≤ *S* < 4.5) and did not change significantly among samples. In contrast, the *S* values of two chromosomes increased from promastigotes to amastigotes, as follows: (i) chromosome 8, from 2.15 ± 0.4 to 3.07 ± 0.12 in P3 and 2.95 ± 0.16 in P4 (both differences significant), and (ii) chromosome 10, from 1.74 ± 0.37 to 2.30 ± 0.10 in P3 (not significant) and 2.47 ± 0.14 in P4 (marginally significant) (Fig. 2C; Data Set S1A1).

**FIG 3:**
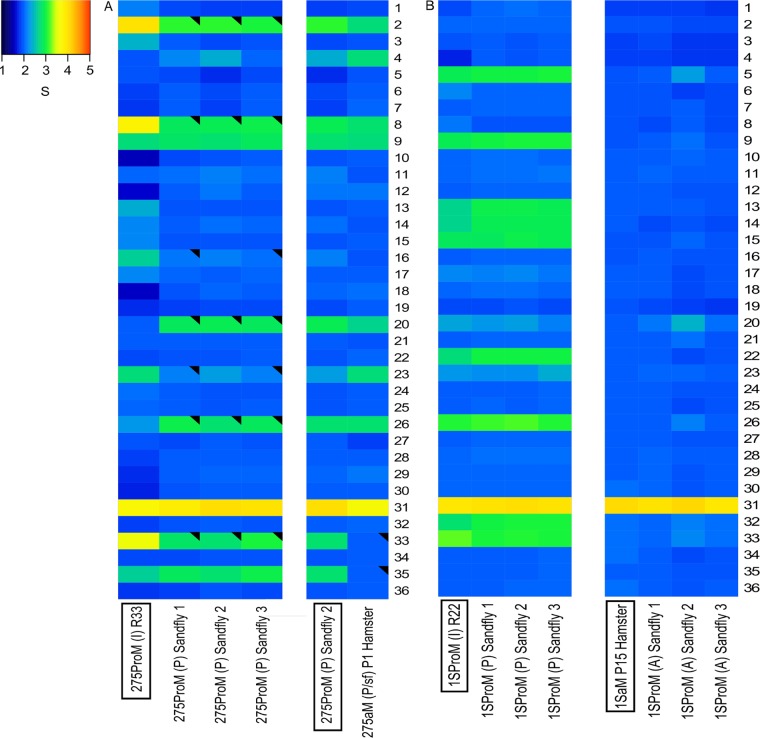
Dynamics of aneuploidy of *L. donovani* BPK275/0 (A) and Ld1S (B) during adaptation to different *in vivo* environments. See legend to Fig. 2 for details. See Data Set S1A1 and A2 for all of the pairwise comparisons.

10.1128/mBio.00599-17.1 DATA SET S1  (A) Statistical significance of changes in *S* values for samples whose results are shown in Fig. 2 and 3 (A1) and for all the samples (A2). In the text and in Fig. 2 and 3, we illustrated the most significant somy changes among samples (in boldface here). (B) Comparison between the *S* value of each chromosome and corresponding RNA-*S* per sample, with corresponding median absolute deviation. (C) Individual gene expression levels in promastigotes showing variable aneuploidy. Transcript depth ratios were computed between samples with trisomic and disomic chromosomes, and median and MAD values calculated per chromosome. All genes presenting a transcript depth ratio of +2.5 MAD (overexpressed outliers) or −2.5 MAD (underexpressed outliers) are listed here. (D) Upregulated genes in promastigotes (D1) and amastigotes (D2). Their gene product and GO terms were listed along with corresponding standard DEseq fold change analysis results. Overexpressed genes were defined as having a fold change of ≥2 and Benjamini-Hochberg-adjusted P value of ≤0.05. Download DATA SET S1, XLSX file, 0.3 MB. Copyright © 2017 Dumetz et al. 2017 Dumetz et al. This content is distributed under the terms of the Creative Commons Attribution 4.0 International license.

10.1128/mBio.00599-17.6 TABLE S3  Quantitative changes of the different karyotypes observed in Fig. 2 and 3 across all of the BPK282 samples (A), BPK275 samples (B), and Ld1S samples (C). Tables A, B, and C summarize the significant changes in *S* values observed during the course of the experiments (raw data are in Dataset S1). Download TABLE S3, PDF file, 0.01 MB. Copyright © 2017 Dumetz et al. 2017 Dumetz et al. This content is distributed under the terms of the Creative Commons Attribution 4.0 International license.

Subsequently, *in vivo*-adapted amastigotes (aM P4) were used (i) to infect sand flies to assess the stability of aneuploidy patterns during transmission (Fig. 2D) and, (ii) after isolation, to follow the karyotype during the short-term adaptation of amastigote-derived promastigotes to *in vitro* culture after 3 and 10 passages, respectively (Fig. 2E). We did not observe any significant karyotype changes during these experiments; we noted a slight decrease in the *S* value of chr10 during early *in vitro* cultivation, from 2.47 ± 0.14 to 1.99 ± 0.12 (marginally significant) (Fig. 2E).

### Karyotype dynamics is strain specific.

Given the observed flexibility in aneuploidy patterns of BPK282/0, we further explored whether this was a specific feature of this particular line or if it could be also found in other *L. donovani* lines. To address this question, we analyzed (i) another uncloned *L. donovani* clinical line from the Indian subcontinent (ISC), BPK275/0 (sodium stibogluconate [SSG] resistant) and (ii) a cloned laboratory strain, Ld1S, isolated in Sudan in 1962 and propagated for this study either *in vitro* for approximately 200 generations or *in vivo* for at least 15 passages in hamsters (Fig. 1B and C; Data Set S1A1).

After the development in sand flies of *in vitro*-adapted promastigotes of BPK275/0 [275ProM (I) R33], we observed that the *S* values of 7 chromosomes consistently changed (Table S3). For example, in sand fly 1, (i) the *S* values of chromosomes 2, 8, and 33 decreased significantly, from 4.00 ± 0.90 to 3.10 ± 0.18, from 3.80 ± 1.05 to 2.89 ± 0.22, and from 3.57 ± 0.92 to 2.82 ± 0.12, respectively, and (ii) the *S* values of chromosomes 16 and 23 decreased significantly, from 2.64 ± 0.56 to 2.10 ± 0.10 and from 2.72 ± 0.50 to 2.17 ± 0.13, respectively, while (iii) the *S* values of chromosomes 20 and 26 increased significantly, from 2.00 ± 0.44 to 2.88 ± 0.16 and from 2.26 ± 0.45 to 2.94 ± 0.14, respectively (Fig. 3A; Data Set S1A1 and Table S3). The aneuploidy patterns in sand fly 3 were identical, while in sand fly 2, 2 of the 7 varying chromosomes (16 and 23) also decreased, but the differences did not reach the threshold of significance (Fig. 3A; Data Set S1A1 and Table S3). Next, we inoculated promastigotes isolated from one fly to hamsters and analyzed the karyotype in the amastigotes isolated 2 months after infection. The *S* values changed as follows: (i) for chromosomes 33 and 35, there were significant decreases, from 2.73 ± 0.23 to 1.97 ± 0.17 and from 2.76 ± 0.19 to 2.00 ± 0.16, respectively, and (ii) for chromosomes 4 and 23, there were increases (marginally significant), from 2.29 ± 0.23 to 2.76 ± 0.25 and from 2.34 ± 0.17 to 2.77 ± 0.21, respectively (Fig. 3A; Data Set S1A1 and Table S3). In contrast, we did not observe major changes in aneuploidy patterns for the Ld1S strain after passage through sand flies, whether *in vitro*-grown promastigotes or hamster-adapted amastigotes were used as the source of infection (Fig. 3B; Table S3 and Data Set S1A).

### Changes in chromosome copy number correlate with overall transcriptome levels.

**FIG 4:**
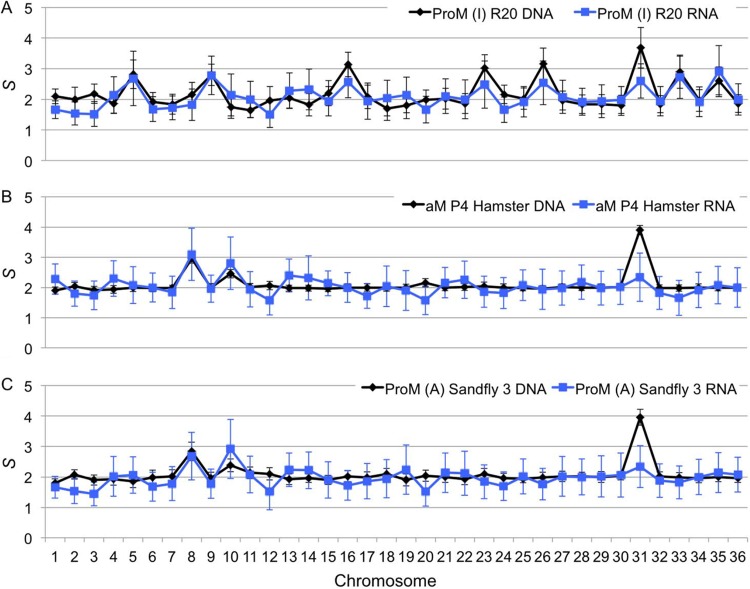
Effects of variable aneuploidy of *L. donovani* BPK282 on transcriptomes during adaptation to different environments. Three life stages/environments in which aneuploidy differs are considered: *in vitro* promastigotes (R20) (A), amastigotes adapted to hamster (P4) (B), and sand fly-derived promastigotes (C). The black and blue lines correspond to *S *values and RNA-*S* values, respectively. The error bars correspond to median absolute deviations. Details on the correlation between *S* and RNA-*S* can be found in Table S4, and the values are in Data Set S1B.

It has been proposed that *Leishmania* organisms use aneuploidy to increase mRNA levels, and we have previously shown a correlation between chromosome copy numbers and transcript levels in one promastigote sample of BPK282/0cl4 (19). Thus, we hypothesized that the observed variation in aneuploidy is potentially adaptive and has a functional effect on the parasite’s biology. Therefore, we would expect that an increase or decrease in chromosome copy number would be mirrored by a proportional change in transcript levels derived from this chromosome. To address this hypothesis, we performed high throughput RNA sequencing (RNA-seq) analysis (detailed in Table S1B) to obtain transcriptome profiles of selected representative samples of BPK282/0 and BPK275/0 for which we previously assessed the aneuploidy patterns (Fig. 1). For each chromosome, we computed the median transcript level (hereinafter referred to as RNA*-S*; see Materials and Methods) and compared this with DNA-based *S* values. From this, we could observe that for all chromosomes except chromosome 31, changes in *S* values were generally mirrored by alterations of RNA-*S* in the same direction, as illustrated in Fig. 4, as well as in Fig. S1A and Data Set S1B. Within each sample, the correlation between *S* and RNA-*S* values, excluding the values for chromosome 31, was significant, and the coefficient of determination *r*^2^ ranged from 0.2 to 0.7 with a *P* value of <0.01, except for ProM (A) R3 (Table S4). Overall, when all samples were combined, we found significant correlations, with (i) an *r*^2^ of 0.395 and a *P* value of 1.05 × 10^−43^ without chromosome 31 and (ii) an *r*^2^ of 0.341 and a *P* value of 1.34 × 10^−37^ when chromosome 31 was included.

10.1128/mBio.00599-17.3 FIG S1  (A) Correlation between *S* and RNA-*S* during adaptation to different life stages/environments. The correlation coefficient between *S* and RNA-*S* in promastigotes (*in vitro* and from sand fly) and amastigotes was *r* = 0.65 (*P* = 7.7 × 10^−12^). Chromosome 31 somy values were excluded for the correlation calculation but were included in the scatter plot to illustrate their behavior. (B) Manhattan plot for individual gene expression in promastigotes showing variable aneuploidy. Samples considered here are ProM (I) R20, ProM (P) sand fly 3, Prom (A) R3, ProM (A) R10. These samples represent the same life stage, but they differ in the somies of chromosomes 5, 8, 9, 16, 23, 26, 33, and 35. RNA depth ratios between trisomic and disomic groups in these chromosomes are shown: the *x* axis represents each coding unit along chromosomes, and the *y* axis the average RNA depth ratios. Colors of dots were alternated for each chromosome. Download FIG S1, PDF file, 0.2 MB. Copyright © 2017 Dumetz et al. 2017 Dumetz et al. This content is distributed under the terms of the Creative Commons Attribution 4.0 International license.

10.1128/mBio.00599-17.7 TABLE S4  Correlation between genome and transcriptome. The values of Pearson correlation coefficient *r*, determinant *r* × *r* (*r*^2^), and its statistical significance between *S* and RNA-*S* are shown. Chromosome 31 was tetrasomic in all samples and was excluded from this analysis because its transcription level was systematically close to the disomic level. Download TABLE S4, PDF file, 0.01 MB. Copyright © 2017 Dumetz et al. 2017 Dumetz et al. This content is distributed under the terms of the Creative Commons Attribution 4.0 International license.

**FIG 5:**
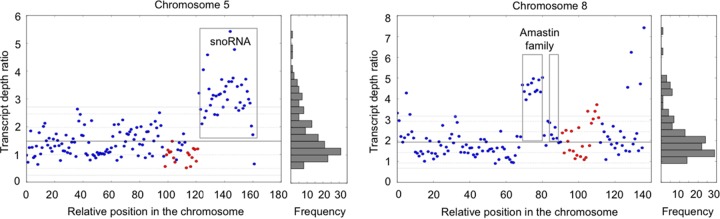
Individual gene expression in promastigotes showing variable aneuploidy. Samples considered here are ProM (I) 20, ProM (P) sand fly 3, ProM (A) R3, and ProM (A) R10. Transcript depth ratios between samples with trisomic and disomic chromosomes 5 and 8 are shown. Each single dot represents a transcript; blue indicates the plus strand, and red the minus strand. The *x* axis represents each coding unit along the chromosome, the *y* axis the average transcript depth ratios, and corresponding histograms show their distribution. The snoRNA cluster on chromosome 5 and the amastin family are marked with boxes. The thick gray horizontal lines indicate the median depth, and the thin gray lines represent 1 MAD (median absolute deviation), 2 MAD, and 2.5 MAD, respectively, away from the median depth.

Although overall the chromosomal transcription levels correlated with the somies of the respective chromosomes, this does not exclude the possibility that the transcription of some specific genes might not follow this overall trend. A potential mismatch between chromosomal and local gene transcription levels was thus checked for samples of a same life stage, in order to minimize the effect of regulation occurring between life stages (see below). We chose 4 promastigote samples of BPK282/0 [ProM (I) 20, ProM (P) sand fly 3, ProM (A) R3, and ProM (A) R10] and focused on chromosomes showing significant changes in *S* values, i.e., chromosomes 5, 8, 9, 16, 23, 26, 33, and 35. For these chromosomes (Fig. S1B), we compared the average transcript depth ratio for each RNA coding unit between the samples with overall trisomic (2.5 ≤ *S* < 3.5) and disomic chromosomes (1.5 ≤ *S* < 2.5) and found 13 to 18% outlier genes in these 8 chromosomes (Table S5 and Data Set S1C). Interestingly, most of these outliers (80%) were overexpressed. The results for chromosomes 5 and 8 called for further attention (Fig. 5). Specifically, we found 40 transcripts at the end of chromosome 5, from LdBPK_050017400 to LdBPK_050021300, which were more highly expressed than other genes in strains with trisomic chromosome 5. Remarkably, the 40 transcripts belong to one transcription unit (starting after a strand switch region), and 38 of them encode snoRNAs: this entire polycistronic unit thus seemed to be regulated independently from the rest of the chromosome. Chromosome 8 in turn showed an array of amastin genes (from LdBPK_080011900 to 13000) whose expression is 4-fold higher in samples with trisomic chromosome 8 than in samples with disomic ones (Fig. 5; Data Set S1C); the array was a small fraction of a large transcription unit, and interestingly, another amastin array (from LdBPK_080013400 to 13800) situated in the same transcription unit was not overexpressed.

10.1128/mBio.00599-17.8 TABLE S5  Proportions of outlier transcripts compared to the *S* value of their chromosomes for chr 5, 8, 9, 16, 23, 26, 33, and 35, Description of the number of transcripts on the chromosomes concerned and the number of transcripts not following the general expression (up or down) of the chromosomes according to their *S* values. Download TABLE S5, PDF file, 0.01 MB. Copyright © 2017 Dumetz et al. 2017 Dumetz et al. This content is distributed under the terms of the Creative Commons Attribution 4.0 International license.

### Individual gene expression in different life stages is characterized by a shared aneuploidy pattern.

**FIG 6:**
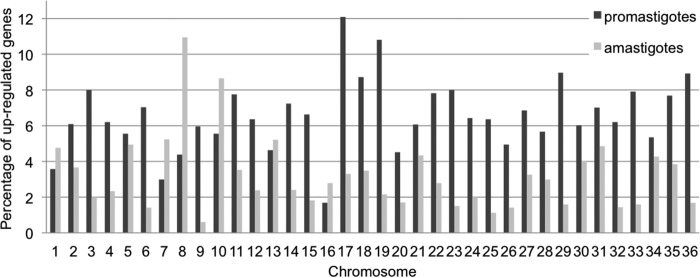
Aneuploidy-independent changes of transcriptomes during parasite differentiation. The samples considered here have the same aneuploidy pattern and correspond to (i) adapted amastigotes of hamsters (aM P3 and P4 taken together) and (ii) promastigotes freshly differentiated from these [ProM(A) R3 and R10 taken together]. The histogram shows the percentage of upregulated coding units that have a ≥2-fold change and Benjamini-Hochberg-corrected *P* value of ≤0.05 in each chromosome based on the DEseq analysis.

It is known that the expression of some *Leishmania* genes is differently regulated from one life stage to the other, over a short period during the life cycle (20). To integrate this, we compared the transcriptomes of two consecutive amastigote samples with those of two *in vitro*-grown promastigotes [aM P3 and aM P4 versus ProM (A) R3 and ProM (A) R10] derived from them. These two amastigote samples originated from two consecutive passages in hamsters, and the promastigotes are derived from the last amastigote passage; thus, we considered each stage-specific pair of samples as biological replicates. Importantly, all 4 of these samples had identical aneuploidy patterns (Fig. 2C and E), allowing the identification of variation in the transcriptome independent of gene dosage. With a cutoff for differential expression (DE) of a 2-fold change with a Benjamini-Hochberg-corrected *P* value of <0.05, we found that 589 genes (6.8%) were upregulated in promastigotes compared with their transcription levels in amastigotes (Data Set S1D1), while only 261 genes (3.0%) were expressed at higher levels in amastigotes (Data Set S1D2) and the remaining 7,831 genes (90.2%) were constitutively expressed. When looking closely at the function of the DE genes in the two life stages, we identified that, in promastigotes, several genes belonged to categories related to carbon, lipid, and fatty acid metabolism, translation, protein modification, membrane transport, DNA replication and nucleosome assembly, and the function of the flagellum (Table S6). We could not find genes belonging to these categories in the upregulated genes in amastigotes. Instead, we found 25 genes coding for amastins and amastin-like proteins to be upregulated in amastigotes and only 5 in promastigotes. We also analyzed the relative distributions of the overexpressed genes over the different chromosomes. We found the highest percentages of upregulated genes on chromosomes 8 and 10 in amastigotes, while chromosomes 17 and 19 contained the highest percentages of genes upregulated in promastigotes (Fig. 6; Data Set S1D1 and D2).

10.1128/mBio.00599-17.9 TABLE S6  GO terms based on biological process for differentially upregulated genes in amastigotes or promastigotes. Genes were considered upregulated if their ratio was ≥2-fold and *P* value was ≤0.05 (DEseq analysis). Amastin, which is not associated with any GO term, was added to the table manually. Download TABLE S6, PDF file, 0.01 MB. Copyright © 2017 Dumetz et al. 2017 Dumetz et al. This content is distributed under the terms of the Creative Commons Attribution 4.0 International license.

## DISCUSSION

Primarily, this study shows that passaging and adaptation of both *in vitro* and *in vivo* clinical lines of *L. donovani* result in little or no variation in SNPs, indels and CNVs, while large changes in aneuploidy occur over short periods of time, such as one passage through a sand fly (12.5 generations, i.e., 2.5 generations/day over a period of 5 days [21]) or a mammalian host (approximately 5 generations, assuming a generation time of 12 days [22] over 2 months). Whether this is due to preferential growth of subpopulations with different genomic variants or *de novo* generation of genomic changes remains to be further investigated. Although our experimental setup included mostly uncloned isolates and thus, potentially, subpopulations of different karyotype, it should be noted that even in clonal populations, individual parasites were reported to differ in their karyotype, based on WGS-based somy measurements in fact representing the population average (13). Mosaicism likely explains the noninteger values that we observed for *S* values of individual samples or for differences between samples. This mosaic aneuploidy is supposed to provide a strong adaptive advantage for the whole population rather than for the single cell (16), with beneficial aneuploidies being selected in response to environmental changes.

Regardless of the mechanisms underlying this genome plasticity, it is likely that such changes in aneuploidy are adaptive, as similar changes have been observed in response to drug pressure or changes in the environment in *Leishmania* organisms during *in vitro* experimental evolution studies, as well as in other eukaryotic pathogens (15, 23, 24). Several indicators of adaptive benefits were observed here. Aneuploidy patterns were mirrored across replicates in the sand fly and also in the transcriptome changes (except for chromosome 31), indicating both a shared adaptation and a potential function of the genomic variation in adjusting gene dosage. Such changes were also found to be progressive. Amastigote adaptation to hamsters resulted in a somy decrease at P1 (chromosome 16) which continued at P3 and P4 (chromosomes 5, 9, 23, and 26). Conversely, increases in somy were observed for chromosomes 8 and 10 upon hamster passaging (Fig. 4). These two chromosomes also had increased regulation of individual genes in amastigotes compared to their expression in promastigotes (Fig. 6). Chromosome 8 contains several copies of δ-amastins (25), genes only found in trypanosomatids that possess an intracellular life stage, in which they are highly expressed (26). RNA interference (RNAi) knockdown experiments have also shown that δ-amastin expression in *Leishmania braziliensis* is needed for the survival and proliferation of intracellular parasites in *in vitro* macrophage infections and in *in vivo* infection of mice (26). Similarly, chromosome 10 contains the *gp63* gene array, a major virulence factor in promastigotes (27) and protection factor in intracellular amastigotes (28). Thus, the increased somies of chromosomes 8 and 10 in hamster-adapted amastigotes may provide adaptive advantages due to increased copy numbers of δ-amastin and *gp63* genes, although further experiments are required to confirm this.

Notably, strain-specific differences were observed here. Like BPK282/0, BPK275/0 also showed a plastic karyotype, but increases in somy were detected in the sand fly (chromosomes 20 and 26) and at an early stage of adaptation to the hamster (chromosomes 4 and 23). In contrast, the karyotype of Ld1S was very stable in the sand fly, and all chromosomes (except 31) were disomic in amastigotes isolated from P15 hamster. This could be explained by the genetic distance of the latter strain, originating from East Africa, although this karyotype stability could be a consequence of its historical *in vivo* laboratory passaging. This strain specificity is in line with our previous phylogenomic study in natural populations of *L. donovani* (promastigotes), where parasites belonging to the same phylogenetic clade showed a higher karyotypic similarity than those from different clades (12). This suggests that the optimal karyotype depends on the genetic background of a particular strain (29), a feature that warrants further investigation in this and other *Leishmania* species.

Although this study addressed the gene dosage-adaptive role of aneuploidy, this strategy may also be beneficial in other areas, such as increasing diversity and heterozygosity, especially given the minimal sexual recombination described in this organism (13). We demonstrated this effect in natural populations of *L. donovani*, where we found that epidemic disomic clones emerging from aneuploid ancestors showed a significantly lower degree of heterozygosity (12). Although this effect could not be studied here due to the scarcity of heterozygous sites in BPK282/0, this phenomenon is being investigated in the Ld1S strain (Prieto Barja P, Pescher P, Bussotti G, Dumetz F, Imamura H, Kedra D, Domagalska M, Chaumeau V, Himmelbauer H, Pagès M, Sterkers Y, Dujardin JC, Notredame C, Späth G, submitted for publication).

Although several life stage comparison studies on short-term transcriptome differentiation have been undertaken previously (30, 31), the potential impact of changes in aneuploidy patterns on transcription profiles has not been studied. Our study clearly shows that life stage-/environment-induced changes in aneuploidy have great impacts on the transcriptome, but at the same time, they do not remove the effect of stage-specific regulation of he transcriptome, which is required during the transition from one stage to the other. While chromosome copy number is mirrored by transcript levels in this study, these should be also reflected at the protein and metabolite levels to have a phenotypic effect. Although genes are constantly transcribed in a polycistronic manner by this process, it is likely that only a few specific genes required for adaptation to a given environment are the main drivers, with the expression levels of other genes regulated via posttranscriptional mechanisms (32). In our data set, we observed that although an overall correlation between the quantities of DNA and RNA exists, not all individual transcripts found on variable chromosomes change to the same extent: this was most obvious in the case of snoRNAs in chromosome 5 and one amastin array in chromosome 8. Chromosome 31 constituted a major exception to the DNA/RNA correlation, as it behaved transcriptionally as a disomic chromosome, despite being tetrasomic. Tracking heterozygous sites and comparing alternative allele frequencies in both DNA and RNA should allow an answer to the question of whether the four chromosomes are transcriptionally active; this was not possible with BPK282/0 because of the lack of heterozygosity in that strain.

*Leishmania* amastigotes are thought to exist in a semiquiescent state with reduced growth, metabolism, and energy consumption (22, 33). This hypothesis is supported here, with several genes being downregulated in amastigotes compared to their expression in promastigotes, primarily those involved in metabolism, nucleosome assembly, and protein assembly and processing (see Table S6 in the supplemental material). Reduced somy was also associated with the amastigote stage in hamsters, where disomy was more common than in *in vitro*-grown promastigotes (Fig. 2) (12). Thus, during *in vitro* growth, when nutrient availability is not limiting, high levels of aneuploidy are allowed, while during the adaptation to semiquiescent state, minimal levels of somy would be required.

Overall, this study highlights the effects of life stage and *in vitro* maintenance on the chromosomal copy numbers of a given strain. This would have a potential impact on molecular epidemiological studies performed on *in vitro*-cultivated promastigotes, as done previously (11, 12). While this effect may be small, some genomic features could be diluted/altered during *in vitro* maintenance, even if this was rather brief (about 20 subinoculations after isolation from the patient). We showed that chromosome copy number is the most sensitive to environmental changes, which possibly explains the lack of correlation between aneuploidy and drug resistance of natural isolates (12), while this is a common feature during experimental selection (14, 15). This highlights the importance of using clinical samples and minimal laboratory passaging when linking genomic adaptations to drug or immune pressure in future studies.

## MATERIALS AND METHODS

### Parasites.

Three *L. donovani* lines were used in this study: the SSG-sensitive and reference strain for *L. donovani* from the Indian subcontinent (ISC) (ISC6 genetic group) (12, 19), MHOM/NP/03/BPK282/0 (BPK282/0) (the derived clone 4 was used for the *L. donovani* reference genome; the latter is also referred to as BPK282A1 [19]); the SSG-resistant strain (34) MHOM/NP/03/BPK275/0 (BPK275/0) (ISC5 genetic group [12]); and strain MHOM/SD/62/1S-CL2D (Ld1S), maintained *in vivo* in hamsters, originally obtained from Henry Murray (Weill Cornell Medical College, New York, NY, USA) (35, 36).

### Experimental models.

The main experiment of this study was performed using the uncloned *L. donovani* line MHOM/NP/03/BPK282/0. The genome sequence and karyotype of the clinical line and the derived clone 4 promastigotes were found to be identical. Genome changes of the BPK282/0 line were followed in a series of *in vitro* and *in vivo* experiments using golden Syrian hamsters and *Phlebotomus argentipes* to mimic the natural vertebrate and invertebrate hosts, respectively, of *L. donovani* in the ISC. All life stages, environments, sample names, and analyses undertaken are outlined in the experimental flowchart in Fig. 1A. In the case of BPK275/0 and Ld1S, the experimental setups were simpler (detailed in Fig. 1B and C); for Ld1S, originating from Sudan, *P. orientalis* was used, as it is the natural sand fly vector of *L. donovani* in East Africa.

Promastigotes were maintained in culture in M199 medium (Gibco) supplemented with 20% (vol/vol) heat-inactivated fetal calf serum (FCS), 4 mM NaHCO_3_, 100 µM adenine, 7.6 mM hemin, pH 7.6, at 26°C for 7 days. For DNA/RNA extraction, promastigotes were harvested in logarithmic phase (day 4) by pelleting for 10 min at 1,700 × *g* at room temperature. Hamsters were infected and amastigotes were purified as described elsewhere (37). Hamsters P1 and P2 were maintained for 3 months each, while hamsters P3 and P4 were maintained until they showed clinical signs of illness in order to recover enough amastigotes to extract DNA and RNA. Parasites were resuspended at 2 × 10^8^ amastigotes per ml in phosphate-buffered saline (PBS) and pelleted for DNA/RNA extraction. Hamster care and experimental procedures were performed with the approval of the Animal Ethics Committee of the Institute of Tropical Medicine Antwerp (approval BM2013-8) and were compliant with the national and international laws for the protection and welfare of animals. The generation of sand fly-derived promastigotes was performed at the secure sand fly insectary at the Department of Parasitology in Prague as previously described by Sadlova et al. (38), with the following modifications: *P. argentipes* (old laboratory colony originating from India) or *P. orientalis* (originating from Ethiopia in 2010) sand flies were fed on blood containing promastigotes or hamster-derived amastigotes. After 5 days, mature infection was reached and 15 *P. argentipes* and 10 *P. orientalis* whole midguts were individually transferred into M199 medium with 10% FCS and fluorocytosine for 5 days. Three reisolates were then pelleted and cryopreserved for DNA/RNA extraction, which was performed using the AllPrep DNA/RNA minikit (Qiagen) following the manufacturer’s instructions. In the case of Ld1S, DNA extractions were performed before cryopreservation with the DNeasy blood and tissue kit from Qiagen.

### Genome and transcriptome sequencing.

PacBio sequencing was used to generate a new LdBPK282 reference genome ftp://ftp.sanger.ac.uk/pub/project/pathogens/Leishmania/donovani/LdBPKPAC2016beta/ (for details, see Text S1 in the supplemental material; also see Appendices A1 to A3 at http://www.itg.be/modaneu), and the European Nucleotide Archive accession number for the PacBio read data is ERP022358; DNA from all other samples was sequenced on Illumina platforms. Briefly, DNA-sequencing libraries were prepared using different methods, as follows. (i) The Nextera XT DNA library prep kit (Illumina, Inc.) was used with 1 ng of input genomic DNA according to the manufacturer’s instructions, and the resulting library quantified by quantitative PCR (qPCR) using a KAPA library quantification kit optimized for the Roche LightCycler 480 (Kapa Biosystems) on a LightCycler 480 (Roche). This was followed by paired-end sequencing (2 × 100 bp) on a HiSeq 1500 platform, producing an average depth of 51× for diploid chromosomes; the European Nucleotide Archive accession number of these Nextera reads is ERP022358. (ii) TruSeq DNA library preparation was performed by shearing genomic DNA into 400- to 600-base-pair fragments (Covaris Adaptive Focused Acoustics technology), followed by generating 125-bp paired-end reads on the HiSeq 2000 version 4 according to the manufacturer’s standard sequencing protocol (39). Raw sequence data were deposited in the European Nucleotide Archive with accession number ERP017317; (iii) for all the Ld1S samples, the libraries were prepared using the KAPA hyper prep kit (Kapa Biosystems) with an input of 0.3 to 0.6 µg of sheared genomic DNA (Covaris E201) according to the manufacturer’s instructions. The final libraries were quantified with the KAPA library quantification kit (Kapa Biosystems). Each library was sequenced in paired-end mode at 2 × 101 bp in a fraction of one sequencing lane of a HiSeq 2000 flow cell version 3 (Illumina, Inc.) according to standard Illumina operation procedures (see Text S1 in the supplemental material for a table specifying which library method was used for which sample).

10.1128/mBio.00599-17.10 TEXT S1  Supplemental materials and methods. Download TEXT S1, DOCX file, 0.03 MB. Copyright © 2017 Dumetz et al. 2017 Dumetz et al. This content is distributed under the terms of the Creative Commons Attribution 4.0 International license.

RNA-seq libraries were prepared using the Illumina TruSeq stranded mRNA sample preparation kit according to the manufacturer’s standard protocol, apart from the PCR amplification, which was performed using KAPA HiFi polymerase. Seventy-five-base-pair paired-end reads were generated on the HiSeq 2000 version 4 according to the manufacturer’s standard sequencing protocol. Raw sequence data were deposited in the European Nucleotide Archive with accession number ERP017437.

### Genome data analysis.

The methods for mapping the reads, SNP and indel calling, and local copy number assessment are described in detail in Text S1.

### Somy estimation.

For somy estimation, we used three different methods based on the depth variability and total read depth of the sample. Somy values were first estimated using the median depth across each chromosome (12, 19), allowing the evaluation of sequencing-quality depth statistics (average values and standard deviations). While this method works optimally for samples with an average depth greater than 20, those whose average is below this threshold were analyzed using a bin-based method. This method splits each chromosome into 2,500-bp bins, and a chromosome-wide median read depth, *dr*_*i*_, is calculated from these bins. The *dr*_*i*_ values are normalized against the median depth of neighboring chromosomes, *dm* (see below), to get the chromosome somy value *S*_*i*_, using the formula *S*_*i*_ = 2 × *dr*_*i*_/*dm*, where *i* is the number of chromosomes (1 … 36). This method has been shown to be more suitable for depths of around 4× coverage and is able to handle larger depth variations than the simple median method (12, 19). For the aM (P/sf) P1 hamster sample, whose mean depth was less than 0.8, the somy was estimated based on the number of reads per 1,000 bp, and median absolute values of its somy values could not be computed.

Previous studies have shown some correlation between chromosome depth and length for some sequencing runs (11, 19). We thus consider this to be caused by sequencing artifacts and applied a bias correction to the estimate of the median depth *dm*. Instead of calculating 1 *dm* based on all chromosomes, this value was calculated based on the median values of neighboring chromosomes, whose lengths are similar since chromosomes are numbered according to increasing size. Thus, somy values *S*_*i*_ were calculated using a *dm* based on the median read depth of 15 chromosomes: the 7 neighboring chromosomes on each side and the chromosome itself. When a chromosome did not have 7 neighboring chromosomes on one side, mirror-imaged values were used, so that some depth values were used twice.

The range of monosomy, disomy, trisomy, tetrasomy, and pentasomy was defined, as previously described (11, 12, 19), to be the full cell-normalized chromosome depth or somy of S, S < 1.5, 1.5 ≤ *S* < 2.5, 2.5 ≤ *S* < 3.5, 3.5 ≤ *S* < 4.5, and 4.5 ≤ *S* < 5.5, respectively. Neighboring median correction was performed for DNA somy values but not for RNA read depth (see discussion of RNA-*S* in “Transcriptome data analysis” below), where a depth bias associated with chromosome length was not observed. To characterize the statistical variation of *S* values based on median values, we used the median absolute deviation, abbreviated as MAD in the text.

Comparison of *S* values between samples was calculated using the Mann-Whitney U test in SciPy with the median depth values for 2,500-bp bins in two samples as the input. We used two criteria for detecting significant differences, biological and statistical, respectively, as follows: (i) *S* values should differ by more than 0.5 with a shift from one somy distribution range to another, for instance from 1.5 to 2.5 to 2.5 to 3.5, together with (ii) a *P* value of ≤10^−5^. Heat maps were created using the heatmap3 package (40) in R (R Development Core Team 2015). In addition to the observed copy number variation and sequence quality, we inspected all the depths along chromosomes using scripts based partly on the python 2D plotting library Matplotlib (41). We remind the reader that sequencing libraries were generated from DNA extracted from a population of cells, so that somy values calculated from sequencing data are averages across the potentially variable somy of these cells. For this reason, somy values may be noninteger values, representing the mean value of a mixed population.

### Transcriptome data analysis.

The amounts of transcripts were quantified by assessing read depth as described previously (12, 19). For each chromosome, the average depth of transcripts was used to compute an RNA-based relative somy value, here called RNA-*S*. The correlations between DNA and RNA depths, namely, between *S* and RNA-*S*, were calculated and visualized with SciPy (42).

Differentially expressed genes were identified using DEseq 1.18.0 (43). Gene depth information (12, 19) was converted to a count of reads per gene, and then DEseq default parameters were used for the calculations except in estimate dispersions, where method=“pooled” and fitType=“local” were used. Library depth normalization was already performed in the somy calculation steps, and therefore, for each sample, we set the normalization factor to 1 during DEseq calculations. We used a fold change cutoff of ≥2 and a Benjamini-Hochberg-adjusted *P* value of ≤0.05 to define differentially expressed (DE) genes. The percentage of DE genes per chromosome is defined as follows: (number of DE genes per chromosome)/(number of total genes per chromosome) × 100.

Gene ontology information was extracted from the gene information file of *Leishmania major* Friedlin (LmjF) (http://tritrypdb.org/common/downloads/release-8.1/LmajorFriedlin/txt/TriTrypDB-8.1_LmajorFriedlinGene.txt), which contains comprehensible genome information for LmjF, including ortholog, annotation, and GO term information. The ortholog information in the file was used to transfer the annotations from LmjF to LdBPKv2.

To identify genes whose transcriptional level changed more than expected based on changes in chromosome somy, we selected chromosomes in which there were only two distinct DNA somy states in samples from a same life stage (here, promastigotes): ProM (I) R20, ProM (P) sand fly 3, ProM (A) R3, and ProM (A) R10. After determining two groups with different somies, we calculated the ratios of all possible pairwise combinations of the two groups and obtained an average value: the denominators were the normalized RNA coding sequence (CDS) depth values of the group with a smaller somy value, and the numerators were the normalized RNA CDS depth values of the group with a higher somy value. The ratios for each CDS were in chromosome position order.

### Accession number(s).

Normalized RNA CDS read counts were deposited to GEO under GenBank accession number GSE97453.

10.1128/mBio.00599-17.2 DATA SET S2  (A) Conversion of LdBPKv1 identification (ID) to LdBPKv2 ID. Given the higher number of annotated genes in LdBPKv2, together with the corrected misassemblies, we had to rename the ID of each gene. Blast similarity information is given, and its E value cutoff was 10^−20^. (B) Ortholog information for selected multiple-copy genes. Ortholog information was generated by companion annotation runs for LdBPKv1 and LdBPKv2 based on the LmjF reference. Download DATA SET S2, XLSX file, 0.6 MB. Copyright © 2017 Dumetz et al. 2017 Dumetz et al. This content is distributed under the terms of the Creative Commons Attribution 4.0 International license.
