# Supplementary material for: Modulation of Aneuploidy in Leishmania donovani during Adaptation to Different In Vitro and In Vivo Environments and Its Impact on Gene Expression
Source: mBio. 2017 May 23;8(3):e00599-17. doi: 10.1128/mBio.00599-17 (PMC5442457; doi:10.1128/mBio.00599-17)
Supplement: TABLE S1 [file mbo003173320st1.pdf]

Supplementary Table S1A

| Sample ID | Sample ID2 | Library preparation method | Analysis | | | Average Depth* | Norm. Std.* |
|---|---|---|---|---|---|---|---|
| | | | Ploidy | SNP | CNV/gene depth | | |
| aM P3 Hamster | BPK282siQ17 | TruSeq | used | used | used | 63.9 | 0.132 |
| aM P4 Hamster | BPK282siR18 | TruSeq | used | used | used | 13.7 | 0.204 |
| ProM (A) R3 | BPK282siT20 | TruSeq | used | used | used | 32.1 | 0.167 |
| ProM (A) R10 | BPK282siA27 | TruSeq | used | used | used | 13.6 | 0.181 |
| ProM (P) Sandfly 1 | BPK282siU21 | TruSeq | used | used | used | 46.2 | 0.143 |
| | BPK282_Fly_P0_1 | Nextera | no | used | used | 45.7 | 0.204 |
| ProM (P) Sandfly 2 | BPK282siV22 | TruSeq | used | used | used | 84.3 | 0.166 |
| | BPK282_Fly_P0_2 | Nextera | no | used | used | 43.5 | 0.219 |
| ProM (P) Sandfly 3 | BPK282siW23 | TruSeq | used | used | used | 35.2 | 0.2 |
| | BPK282_Fly_P0_3 | Nextera | no | used | used | 60.3 | 0.201 |
| ProM (A) Sandfly 1 | BPK282_Ama_to_fly-1 | Nextera | used | used | used | 106 | 0.202 |
| | BPK282siX24 | TruSeq | no | used | no | 4.7 | 0.552 |
| ProM (A) Sandfly 2 | BPK282_Ama_to_fly-2 | Nextera | used | used | used | 65.4 | 0.215 |
| | BPK282siY25 | TruSeq | no | used | no | 8.2 | 0.412 |
| ProM (A) Sandfly 3 | BPK282_Ama_to_fly-3 | Nextera | used | used | used | 72.5 | 0.2 |
| | BPK282siZ26 | TruSeq | no | used | no | 9.9 | 0.49 |
| ProM (I) R20 | BPK282siP16 | TruSeq | used | used | used | 65.3 | 0.375 |
| ProM (I) R22 | BPK282siS19 | TruSeq | used | used | used | 16.3 | 0.489 |
| | BPK282_Pro_B_fly | Nextera | no | used | used | 63 | 0.347 |
| aM (P/sf) P1 Hamster | BPK282siB28 | TruSeq | used | used | no | 2.2 | 0.13 |
| 275ProM (I) R33 | BPK275si2A1 | TruSeq | used | used | used | 16.2 | 0.54 |
| | BPK275_Pro_B_fly | Nextera | no | used | used | 13 | 0.53 |
| 275ProM (P) Sandfly 1 | BPK275si2B2 | TruSeq | used | used | used | 52.5 | 0.16 |
| | BPK275_Fly_P0_1 | Nextera | no | used | no | 33.3 | 0.224 |

| | | | | | | | |
|---|---|---|---|---|---|---|---|
| 275ProM (P) Sandfly 2 | BPK275si2C3 | TruSeq | no | used | no | 13 | 0.347 |
| | BPK275_Fly_P0_2 | Nextera | used | used | used | 81.2 | 0.19 |
| 275ProM (P) Sandfly 3 | BPK275si2D4 | TruSeq | used | used | used | 36.5 | 0.16 |
| | BPK275_Fly_P0_3 | Nextera | no | used | no | 54.4 | 0.232 |
| 275aM (P/sf) P1 Hamster | BPK275si2E5 | TruSeq | used | used | no | 4.3 | 0.414 |
| 1SaM P15 Hamster | Ld1S_ama | KAPA Hyper Prep Kit | used | no | no | 68.5 | 0.224 |
| 1SProM (A) Sandfly 1 | Ld1S_amasf2 | KAPA Hyper Prep Kit | used | no | no | 109.8 | 0.203 |
| 1SProM (A) Sandfly 2 | Ld1S_amasf5 | KAPA Hyper Prep Kit | used | no | no | 110 | 0.204 |
| 1SProM (A) Sandfly 3 | Ld1S_amasf6 | KAPA Hyper Prep Kit | used | no | no | 74.6 | 0.239 |
| 1SProM (I) R22 | Ld1S_p22 | KAPA Hyper Prep Kit | used | no | no | 81.4 | 0.204 |
| 1SProM (P) Sandfly 1 | Ld1S_p22sf5 | KAPA Hyper Prep Kit | used | no | no | 125 | 0.194 |
| 1SProM (P) Sandfly 2 | Ld1S_p22sf7 | KAPA Hyper Prep Kit | used | no | no | 129.6 | 0.194 |
| 1SProM (P) Sandfly 3 | Ld1S_p22sf11 | KAPA Hyper Prep Kit | used | no | no | 135.5 | 0.182 |

*=These values are based on depth without binning to estimate overall sequence quality.

Supplementary Table S1B

| Sample ID | Sample ID2 | Total (million) | Mapped (million) | (%) | Mapped in pair (million) | (%) | Singletons (million) | (%) | Median depth* | Average depth* | Norm. depth standard deviation* |
|---|---|---|---|---|---|---|---|---|---|---|---|
| ProM (I) R20 | BPK282RNAsiA1 | 36 | 35.29 | 98 | 34.67 | 96.3 | 0.62 | 1.7 | 42.9 | 49.6 | 0.69 |
| aM P3 Hamster | BPK282RNAsiB2 | 38.15 | 37.31 | 97.8 | 36.63 | 96 | 0.68 | 1.8 | 46.0 | 52.8 | 0.70 |
| aM P4 Hamster | BPK282RNAsiC3 | 18.1 | 16.31 | 90.1 | 15.81 | 87.3 | 0.5 | 2.8 | 16.3 | 19.9 | 0.76 |
| ProM (A) R3 | BPK282RNAsiE5 | 35.4 | 34.66 | 97.9 | 33.98 | 96 | 0.67 | 1.9 | 35.6 | 44.2 | 0.79 |
| ProM (A) R10 | BPK282RNAsiF6 | 30.9 | 30.22 | 97.8 | 29.6 | 95.8 | 0.62 | 2 | 29.7 | 38.0 | 0.83 |
| ProM (P) Sandfly 3 | BPK282RNAsiG7 | 30.12 | 29.41 | 97.6 | 28.9 | 96 | 0.51 | 1.7 | 33.9 | 40.6 | 0.74 |
| aM (P/sf) P1 Hamster | BPK282RNAsiH8 | 39.82 | 37.19 | 93.4 | 36.55 | 91.8 | 0.64 | 1.6 | 43.2 | 50.6 | 0.73 |
| ProM (A) Sandfly 3 | BPK282RNAsiI9 | 16.25 | 12.32 | 75.8 | 12.02 | 74 | 0.3 | 1.8 | 6.1 | 8.0 | 0.83 |
| 275ProM (I) R33 | BPK275RNAsiA1 | 33.15 | 32.51 | 98.1 | 31.95 | 96.4 | 0.57 | 1.7 | 42.5 | 47.8 | 0.67 |
| 275ProM (P) Sandfly 2 | BPK275RNAsiB2 | 34.79 | 34.11 | 98 | 33.53 | 96.4 | 0.58 | 1.7 | 41.0 | 48.0 | 0.73 |
| 275aM (P/sf) P1 Hamster | BPK275RNAsiC3 | 18.6 | 16.35 | 87.9 | 16.06 | 86.4 | 0.29 | 1.5 | 20.8 | 24.0 | 0.70 |
| | Total mapped reads | 331.3 | 315.7 | | | | | | | | |
| | Average mapped reads | 30.1 | 28.7 | | | | | | | | |

*=These values are based on depth without binning to estimate overall sequence quality.

Read length =75bp
