# Supplementary material for: Modulation of Aneuploidy in Leishmania donovani during Adaptation to Different In Vitro and In Vivo Environments and Its Impact on Gene Expression
Source: mBio. 2017 May 23;8(3):e00599-17. doi: 10.1128/mBio.00599-17 (PMC5442457; doi:10.1128/mBio.00599-17)
Supplement: TABLE S2 [file mbo003173320st2.pdf]

		aM P3 Hamster	aM P4 Hamster	ProM (A) R3	ProM (A) R10	ProM (P) Sandfly 1	ProM (P) Sandlfy 2	ProM (P) Sandfly 3	ProM (A) Sandfly 1	ProM (A) Sandfly 2	ProM (A) Sandfly 3
Norm std		0.132	0.204	0.167	0.181	0.143	0.166	0.200	0.202	0.215	0.200
DEPTH		63.92	13.705	32.13	13.585	46.195	84.31	35.23	106.04	65.43	72.51
Library prep									N	N	N
H-locus	LdBPK_230007600: hypotheticalprotein,conserved	2.877	2.643	3.281	3	2.875	2.882	2.815	3.213	3.329	3.143
	LdBPK_230007700: terbinafineresistancelocusprotein_yip1_	3	2.571	2.781	2.652	2.694	2.669	2.741	2.567	2.572	2.766
	LdBPK_230007800: pentamidineresistanceprotein1	2.862	2.786	3.062	2.783	2.625	2.803	2.63	2.779	2.814	2.636
	LdBPK_230007900: argininosuccinatesynthase,putative	3.031	2.786	2.812	2.522	2.597	2.834	2.407	3.089	3.429	3.26
	Average for H-locus	2.9425	2.6965	2.984	2.73925	2.69775	2.797	2.64825	2.912	3.036	2.95125
M-locus	LdBPK_360076600: tartrate-sensitiveacidphosphataseacp-3.2,putative	3.892	4.643	4.367	4.727	4.273	5.458	4.625	6.699	7.622	6.708
	LdBPK_360076700: hypotheticalprotein,conserved	8.092	8.143	7.433	7.409	7.364	8.688	9.75	6.265	6.228	6.333
	LdBPK_360076800: mitogen-activatedproteinkinase	15.661	15.072	13.933	15.319	14.5	12.417	14.937	14.803	15.258	15.568
	LdBPK_360076900: histidinesecretoryacidphosphatase,putative	13.323	13.072	13.6	12.955	13.023	17.632	13.937	12.86	13.91	12.694
	LdBPK_360077000: hypotheticalprotein,conserved	7.538	7.786	7.233	7.591	7.932	8.093	9.937	6.133	6.319	6.444
	Average for M-locus	9.7012	9.7432	9.3132	9.6002	9.4184	10.4576	10.6372	9.352	9.8674	9.5494
Ch6 - Yeti amplicon	LdBPK_060014000: hypotheticalprotein,conserved	1.016	0.923	1.061	1.136	0.896	0.989	0.778	0.971	1	0.829
	LdBPK_060014100: hypotheticalprotein,conserved	0.984	0.923	1.03	1	0.917	1.119	0.944	0.853	0.839	0.843
	LdBPK_060014200: dihydrofolatereductase-thymidylatesynthase	0.984	0.769	0.909	0.955	0.937	0.989	0.944	0.961	0.968	0.986
	LdBPK_060014300: arginineN-methyltransferase,typeIII,putative	1.081	0.923	1.061	1	1.021	0.891	1.056	0.951	0.952	0.914
	LdBPK_060014400: acyl-coenzymeadehydrogenase,putative	0.871	0.846	0.849	0.955	0.812	0.848	0.778	0.922	1	0.943
	Average for Yeti amplicon	0.9872	0.8768	0.982	1.0092	0.9166	0.9672	0.9	0.9316	0.9518	0.903

Table S2A

Chr	Ld24		Ld32		Ld32		Ld35		Ld35	
Gene ID	LdBPK_240007400.1		LdBPK_320042200.1		LdBPK_320005900.1		LdBPK_350008700.1		LdBPK_350045600.1	
SNP position on the chromosome	Ld24:65742		Ld32:1398170		Ld32:32369		Ld35:108596		Ld35:1591295	
Protein	hypothetical, con		hypothetical, con		NLI interacting factor-like phosphatase		ATP-dependent DEAD-box RNA helicase%2C putative		4E-interacting protein	
Changes (REF/ALT)	T/G => Stop		C/G=> Ala/Gly		A/G => Cys/Arg		C/T => Ser/Phe		A/T => Stop	
	DNA seq	RNA seq	DNA seq	RNA seq	DNA seq	RNA seq	DNA seq	RNA seq	DNA seq	RNA seq
ProM (I) R22	8/0	-	13/0	-	2/0	-	5/4	-	16/0	-
ProM (P) Sandfly 1	39/0	-	56/0	-	52/0	-	41/0	-	49/0	-
ProM (P) Sandfly 2	65/0	-	95/0	-	44/0	-	66/0	-	108/0	-
ProM (P) Sandfly 3	16/0	6/0	13/0	43/0	14/0	98/0	17/0	260/1	14/0	22/0
ProM (P) Sandfly 3	16/0	6/0	13/0	43/0	14/0	98/0	17/0	260/1	14/0	22/0
aM (P/sf) P1 Hamster	2/0	14/0	./.	60/0	2/0	96/0	2/0	303/0	./.	25/0
ProM (I) R20	64/0	13/0	79/0	56/0	37/0	108/0	70/31	329/175	151/0	58/0
aM P3 Hamster	20/35	11/7	29/33	30/32	32/32	58/69	27/28	178/148	28/13	5/5
aM P4 Hamster	7/11	4/4	7/6	10/7	7/12	13/18	3/7	46/49	9/5	4/4
aM P4 Hamster	7/11	4/4	7/6	10/7	7/12	13/18	3/7	46/49	9/5	4/4
ProM (A) Sandfly 4	3/0	-	0/1	-	0/1	-	./.	-	0/2	-
ProM (A) Sandfly 5	2/0	-	1/2	-	2/2	-	1/0	-	2/2	-
ProM (A) Sandfly 6	5/1	1/1	2/6	2/2	1/1	3/10	5/2	26/25	4/3	0/3
aM P4 Hamster	7/11	4/4	7/6	10/7	7/12	13/18	3/7	46/49	9/5	4/4
ProM (A) R3	23/16	5/4	19/19	30/27	14/15	56/86	15/11	192/198	14/16	13/6
ProM (A) R10	8/10	3/3	19/8	20/22	8/10	58/74	8/7	137/134	11/10	9/7

Table S2B
