# Supplementary material for: Modulation of Aneuploidy in Leishmania donovani during Adaptation to Different In Vitro and In Vivo Environments and Its Impact on Gene Expression
Source: mBio. 2017 May 23;8(3):e00599-17. doi: 10.1128/mBio.00599-17 (PMC5442457; doi:10.1128/mBio.00599-17)
Supplement: TABLE S3 [file mbo003173320st3.pdf]

BPK282									
Sample	ProM (P) Sandfly 1	ProM (P) Sandfly 2	ProM (P) Sandfly 3	aM (P/sf) P1 Hamster	aM P3 Hamster	aM P4 Hamster	ProM (A) Sandfly 1	ProM (A) Sandfly 2	ProM (A) Sandfly 3
Compare to	ProM (I) R22			ProM (P) Sandfly 3	ProM (I) R20		aM P4 Hamster		
Total number of chromosome	2	2	2	1	8	8	0	0	0
- Increasing S-value	0	0	0	0	1 (8)	1 (8)	-	-	-
- Decreasing S-value	2 (33,35)	2 (33,35)	2 (33,35)	1 (16)	7 (5, 9, 16, 23, 26, 33, 35)	7 (5, 9, 16, 23, 26, 33, 35)	-	-	-

Sample	ProM (A) R3	ProM (A) R10
Compare to	aM P4 Hamster	
Total number of chromosome	0	0
- Increasing S-value	-	-
- Decreasing S-value	-	-

BPK275					Ld1S					
Sample	275ProM (P) Sandfly 1	275ProM (P) Sandfly 2	275ProM (P) Sandfly 3	275aM (P/sf) P1 Hamster	1SProm (P) Sandfly 1	1SProm (P) Sandfly 2	1SProm (P) Sandfly 3	1SProM (A) Sandfly 1	1SProM (A) Sandfly 2	1SProM (A) Sandfly 3
Compare to	275ProM (I) R33			275ProM (P) Sandfly 2	1SProM (I) R22			1SaM P15 Hamster		
Total number of chromosome	7	5	7	2	0	0	0	0	0	0
- Increasing S-value	2 (20, 26)	2 (20, 26)	2 (20, 26)	0	-	-	-	-	-	-
- Decreasing S-value	5 (2, 8, 16, 23, 33)	3 (2, 8, 33)	5 (2, 8, 16, 23, 33)	2 (33, 35)	-	-	-	-	-	-
