# Supplementary material for: Modulation of Aneuploidy in Leishmania donovani during Adaptation to Different In Vitro and In Vivo Environments and Its Impact on Gene Expression
Source: mBio. 2017 May 23;8(3):e00599-17. doi: 10.1128/mBio.00599-17 (PMC5442457; doi:10.1128/mBio.00599-17)
Supplement: FIG S1 [file mbo003173320sf1.pdf]

S1A

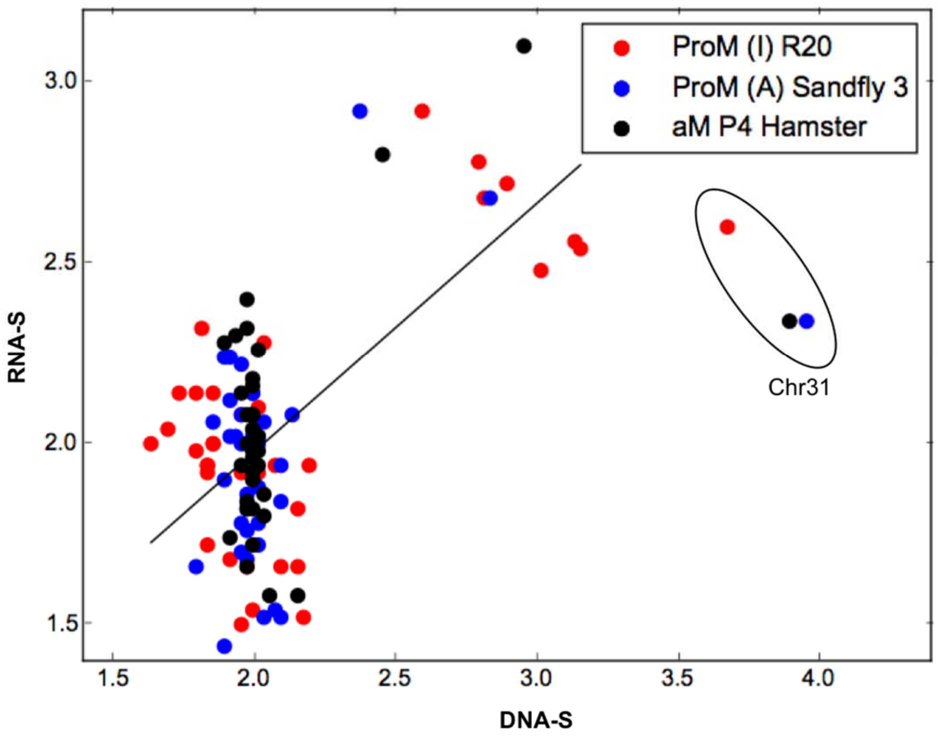

S1B

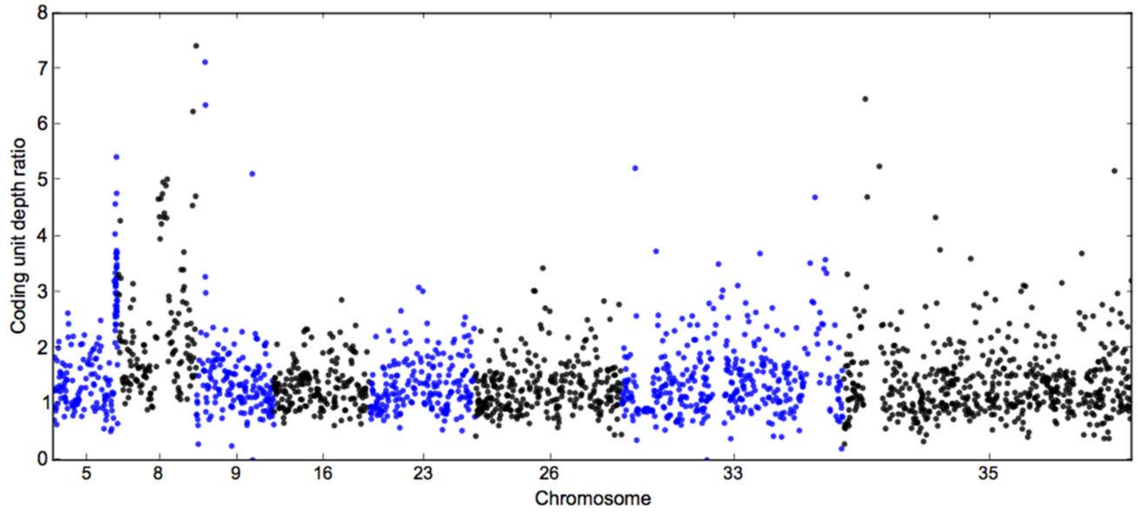

Figure S1. A. Correlation between *S* and *RNA-S* during adaptation to different life stages/environments The correlation coefficient between *S* and *RNA-S* in promastigotes (in vitro and from sand fly) and amastigotes was r =0.65 (p-value $7.7*10^{-12}$). Chromosome 31 somy values were excluded for the correlation calculation but were included in the scattering plot to illustrate their behaviour.

B. Manhattan plot for Individual gene expression in promastigotes showing variable aneuploidy. Samples considered here are: ProM (I) 20, ProM (P) Sandfly 3, Prom (A) R3, ProM (A) R10. These samples represent the same life stage, but they differ in somy of chromosomes: 5, 8, 9, 16, 23, 26, 33 and 35. RNA depth ratios between trisomic and disomic groups in these chromosomes are shown: the x-axis represents each coding unit along chromosomes and the y-axis the average RNA depth ratios. Colours of dots were alternated for each chromosome.
