# Supplementary material for: Modulation of Aneuploidy in Leishmania donovani during Adaptation to Different In Vitro and In Vivo Environments and Its Impact on Gene Expression
Source: mBio. 2017 May 23;8(3):e00599-17. doi: 10.1128/mBio.00599-17 (PMC5442457; doi:10.1128/mBio.00599-17)
Supplement: TABLE S4 [file mbo003173320st4.pdf]

		Length	r	r*r	P-v
275ProM (I) R33	275ProM (I) R33 RNA	35	0.6166	0.3802	8.00E-05
275ProM (P) Sandfly 2	275ProM (P) Sandfly 2 RNA	35	0.6384	0.4076	3.68E-05
275aM (P/sf) P1	275aM (P/sf) P1 RNA	35	0.818	0.6691	1.98E-09
ProM (I) R20	ProM (I) R20 RNA	35	0.668	0.4462	1.16E-05
aM P3 Hamster	aM P3 Hamster RNA	35	0.6694	0.448	1.10E-05
aM P4 Hamster	aM P4 Hamster RNA	35	0.6508	0.4235	2.31E-05
ProM (A) R3	ProM (A) R3 RNA	35	0.2569	0.066	1.36E-01
ProM (A) R10	ProM (A) R10 RNA	35	0.447	0.1998	7.10E-03
ProM (P) Sandfly 3	ProM (P) Sandfly 3 RNA	35	0.6954	0.4836	3.54E-06
aM (P/sf) P1	aM (P/sf) P1 RNA	35	0.7292	0.5317	6.77E-07
ProM (A) Sandfly 3	ProM (A) Sandfly 3 RNA	35	0.5197	0.2701	1.38E-03
