# Supplementary material for: Modulation of Aneuploidy in Leishmania donovani during Adaptation to Different In Vitro and In Vivo Environments and Its Impact on Gene Expression
Source: mBio. 2017 May 23;8(3):e00599-17. doi: 10.1128/mBio.00599-17 (PMC5442457; doi:10.1128/mBio.00599-17)
Supplement: TABLE S5 [file mbo003173320st5.pdf]

Chromosome	All coding units*	Outliers	% of outliers	Up	Down
Ld05	162	27	17%	27	0
Ld08	137	23	17%	22	1
Ld09	168	26	15%	18	8
Ld16	177	31	18%	22	9
Ld23	200	25	13%	22	3
Ld26	283	38	13%	29	9
Ld33	378	56	15%	46	10
Ld35	546	98	18%	73	25

*=All coding units whose depth ratio is defined.
