# Supplementary material for: Modulation of Aneuploidy in Leishmania donovani during Adaptation to Different In Vitro and In Vivo Environments and Its Impact on Gene Expression
Source: mBio. 2017 May 23;8(3):e00599-17. doi: 10.1128/mBio.00599-17 (PMC5442457; doi:10.1128/mBio.00599-17)
Supplement: TABLE S6 [file mbo003173320st6.pdf]

	Up-regulated in promastigotes	Up-regulated in amastigotes
GO annotation with p-value <0.05	372/589 (63.15%)	91/261 (34.9%)
Acid phosphatase	4 (0.68%)	0
ATP binding	3 (0.51%)	0
ATP metabolism	17 (2.88%)	0
Heat-shock protein binding	0	2 (0.76%)
Heme binding	5 (0.85%)	0
Lipid	11 (1.87%)	1 (0.38%)
- Ergosterol + Phospholipid	4 (0.68%)	0
- Fatty acid metabolism	3 (0.51%)	0
- Lipid metabolism	3 (0.51%)	1 (0.38%)
L-methionine salvage	4 (0.68%)	0
Metabolic process	16 (2.72%)	0
Microtubule	9 (1.53%)	0
Nucleic acid	17 (2.89%)	19 (7.28%)
- DNA binding	3 (0.51%)	0
- DNA repair	0	1 (0.38%)
- DNA replication	3 (0.51%)	1 (0.38%)
- RNA binding	11 (1.87%)	13 (5%)
- RNA processing	0	4 (1.53%)
Nucleosome assembly	15 (2.55%)	1 (0.38%)
Oxidation-Reduction	7 (1.19%)	2 (0.76%)
Protein	80 (13.6%)	9 (3.45%)
- Protein folding	27 (4.6%)	1 (0.38%)
- Protein binding	11 (1.88%)	5 (1.91%)
- Post translational modification	10 (1.7%)	3 (1.15%)
- Proteasome	10 (1.7%)	0
- Proteolysis	22 (3.74%)	0
Response to ROS	9 (1.53%)	0
Sugar	14 (2.38%)	0
- Carbohydrate metabolism	10 (1.7%)	0
- Glucose metabolism	3 (0.51%)	0
- Gluconeogenesis	1 (0.17%)	0
Transcription	0	3 (1.15%)
Translation	17 (2.89%)	5 (1.91%)
Transport	9 (1.53%)	4 (1.53%)
