# Supplementary material for: Modulation of Aneuploidy in Leishmania donovani during Adaptation to Different In Vitro and In Vivo Environments and Its Impact on Gene Expression
Source: mBio. 2017 May 23;8(3):e00599-17. doi: 10.1128/mBio.00599-17 (PMC5442457; doi:10.1128/mBio.00599-17)
Supplement: TEXT S1 [file mbo003173320s1.docx]

Supplementary Methods:

***PacBio sequencing and assembly of L. donovani BPK282A1 reference genome***

The reference strain BPK282A1 previously sequenced in 2011 (11) (known as LdBPKv1) was re-sequenced using SMRT WGS (P5C3 chemistry) from Pacific Bioscience RSII. Reads were assembled with the PacBio *de novo* assembly program RS_HGAP Assembly.2.1.0. This produced 616,963 post-filtered reads, a polymerase read N50 value of 11,717 bp, polymerase read length of 8,488 bp, polymerase read quality of 0.836. These reads were assembled and refined into 130 contigs with median 131-fold coverage using default parameters. The sufficient coverage over 100 made it possible for RS_HGAP to perform base error corrections. These contigs were assembled by a succession of methods summarized in Appendix 1; for chromosome building, they were aligned against the *L. major* genome release-8.1 (6).

For ABACAS assembly, the key parameters used were p=nucmer, minimum percent identity 15, minimum contig coverage 15. These relaxed parameters are suitable for very long PacBio contigs. Contigs that were not mapped along were found to be duplicates of the main assembly and un-assembled contigs were not used for further analysis.

PBjelly PBSuite_14.6.24 was applied for gap correction of the contigs using the PacBio reads. This was followed by two rounds of base correction using Quiver. Eight iterations of iCORN v0.97 using Illumina reads from BPK282A1 fixed 70 base errors, 711 insertions, 81 deletions and identified no heterozygous sites. This step was crucial to fix insertions and deletion errors for which Illumina reads were more suitable. Gene annotation was performed using the command line version of Companion v1.0.1, which carried out both *de novo* gene prediction and gene annotation transfer from *L. major* Friedlin (29). The final product of these processes was named LdBPKv2. Annotations of kDNA maxi-circles, which corresponds to Ld37, and ribosomal DNA in chromosome 27, on chromosome 2, were performed by RATT (30), since the initial Companion annotation did not annotate these regions properly.

We used the results of REAPR (31) and Companion to evaluate the relative accuracy of genome assembly among LdBPKv1, LdBPKv2 (*L. donovani*), LinJPCM5 (*L. infantum*) and LmjF reference. It was not optimal to evaluate the quality of PacBio assembly whose N50 is over 11,000 bp with REAPR by using Illumina reads with short insert size. Nevertheless, REAPR provided objective evaluation of the relative accuracy of the Leishmania genomes and the summary of the results of REAPR and Companion were given in Appendix 2. Examples of improvements are mentioned in Appendix 3: (A) Extension of the chromosome sequence up to the telomeres; (B) Increased number of annotated genes, here 18 additional genes in chromosome 27, that were previously missed in LdBPKv1; (C) mapping of tandem arrays, here the mini-exon locus; (D) complete kDNA maxi-circle. Data Set S2A provides the conversion table from LdBPKv1 to LdBPKv2 and Data set S2B, the ortholog information for selected multi copy genes.

Gaps present in LdBPKv2 were mostly introduced during ABACAS assembly and comparisons to other reference indicated that most of large gaps were over estimated. At the time of writing, the improvement of various kinetoplastids genomes is underway, and therefore we did not pursue the further improvement of the genome since it would not affect our results.

***Read mapping SNP and small insertions/deletions detection***

Reads were mapped to the reference L. donovani genome LdBPKv2 using Smalt v7.4 (http://www.sanger.ac.uk/science/tools/smalt-0). Smalt options for exhaustive searching for optimal alignments and random mapping of multiple hit reads were used. Picard v1.85 (http://broadinstitute.github.io/picard/) were used for merging and sorting bam files and marking duplicated reads. This procedure was similar to one described in the previous analysis (12).

SNPs and small insertions and deletions (indels) were called using population-based Unified Genotyper method in Genome Analysis  Toolkit  v3.4 (GATK: <https://software.broadinstitute.org/gatk/>). SNP clusters (more than 3 SNPs within 10 bases of each other) were noted but we maintained these SNPs for analysis. Low quality SNPs were filtered using GATK Variant Filtration with QD < 2.0 || MQ < 40 || FS > 60.0 || ReadPosRankSum < -8.0. To avoid false negatives, the SNP quality cut off was set to 300. Afterwards all candidate SNPs were visually inspected in the Integrative Genomic Viewer (IGV_2_3_47) (33) and SAMtools to avoid false positives. SnpEff v4.1 (34) was used to classify all SNPs and indels based on their functional impact such as frame shift, non-synonymous, synonymous change and intergenic mutation. SNPs and indels were compiled in a population genetic variation variant calling format (vcf ) file. From this vcf file alternative allele and depth information was extracted for further analysis. To ensure the accuracy of variant calling, we also used the base variation data published in previous reports (7, 11, 15). Variants common to all strains were excluded from analysis since our focus was to identify genetic variants different among the samples and these variants are generally associated with technical errors.

***Local copy number variation detection***

Normalized read-depth per haploid genome (haploid depth d) was defined as a raw depth (d_r_), divided by the median depth of its corresponding chromosome (d_ch_), and then d is defined as d_r_/d_ch_ (see 11 and 12). Here depth of each position was measured instead of binned depth used elsewhere in the paper.
